# Innovative psychiatry medical education initiative: empowering and supervising trainees for future teaching in psychiatry training program establishment in Somaliland

**DOI:** 10.1192/bjo.2021.400

**Published:** 2021-06-18

**Authors:** Jibril Handuleh, Victor Periera-Sanchez, Daniel Fekadu Wolde-Giorgis

**Affiliations:** 1 Lecturer in Psychiatry, Amoud University School of Medicine, Psychiatry resident, Saint Paul's Hospital Millennium Medical College; 2 Child and Adolescent Psychiatry fellow NYU Grossman School of Medicine, Visiting lecturer in psychiatry, Amoud University School of Medicine; 3 Head of department, Visiting Professor of psychiatry, Amoud University School of Medicine, Lecturer in Psychiatry, Institute of Psychiatry, Neurology and Psychology, King's College London

## Abstract

**Aims:**

Somaliland is a de facto state in the horn of Africa. It unilaterally declared independence from rest of Somalia in 1991. Medical education in Somaliland started in the year 2000.

Aim of the study is to explore the feasibility of teaching program for the country by its future potential psychiatry educators. The initiative started in 2019 to seek trainees with interest in academic psychiatry and support them with medical education skills. This is intended to prepare them for leading future teaching roles in both undergraduate and residency/fellowship in psychiatry

Amoud University wanted to empower junior doctors at the university to have teaching skills needed to set up residency program. The Somaliland government asked Ethiopian ministry of health to offer psychiatry residency program for general practitioners in Somaliland to have future residency and fellowship in psychiatry. Several psychiatry trainees worked with the visiting professor from the United Kingdom who joined Somaliland medical school as visiting professor in psychiatry

**Method:**

The visiting professor supported the trainee in setting up a psychiatry undergraduate training curriculum in line with Somaliland medical school curriculum. Before the teaching methods were didactic and role play based. The faculty introduced different teaching methods including flipchart, small/large group teaching which was student centered education. Students received a online survey to reflect on psychiatry teaching they received. post course survey was conducted at the end of the teaching to evaluate the teaching initiative.

**Result:**

Survey revealed interesting pattern that students preferred class room based teaching in comparison to online teaching. 90 percent of the attendees showed interest in flipchart teaching compared to didactic model. They expressed increasing understanding of the subject matter when they read and discuss among themselves instead of lectures. 70% of students prefer more clinical teaching compared to online sessions.

52% liked the new teaching module compared to the lecturing sytle.

**Conclusion:**

Supervision of early career psychiatrists to undertake future academic psychiatry roles is an important step in building psychiatry faculty in medical schools. As the case of Somaliland this retains trainees in teaching roles in the future to teach undergraduates mental health courses. The other benefit is empowering them to set up psychiatry training program to close the service delivery gap with skilled psychiatrists in the future. Somaliland plans to set up its psychiatry residency/fellowship programs soon after this initiative.

